# Electricity in General Practice

**Published:** 1909-06

**Authors:** Herbert P. Tayler


### ELECTRICITY IN GENERAL PRACTICE.

BY

HERBERT P. TAYLER, M.A., M.B. Cantab.

---

ELECTRICITY does not receive in general practice the attention that its therapeutic value deserves, owing, no doubt, to the idea that electro-therapeutics require costly and complicated apparatus. This idea may be true if one is to include X-ray photography and high-frequency treatment; but a very great amount can be done for the relief and cure of patients by means of a simple battery of 18 to 20 Leclanché cells, and a small coil driven by a single dry cell to supply the interrupted current.

If, in addition, it is possible to install a Wimshurst static machine, the scope of the work done can be much enlarged. A Wimshurst machine, however, needs a large room to contain it, and almost necessarily an electric motor to drive it; it is also rather a costly instrument, but it lights an X-ray tube perfectly for all screen work, so that it is useful for that, as well as for therapeutic use.

Electricity has been brought before the general public in a marked manner by the discovery of X-rays by Röntgen, and by the advent of the motor-car, so that people talk glibly about matters of which a few years ago they would have confessed complete ignorance; and either thanks to, or in spite of, the half-penny Press, expect to obtain great results from electrical treatment.

The use of electric ignition on motor-cars has led to all sorts of improvements in the manufacture of small accumulators, and the advantage is inestimable of having always available on the car the means of lighting a lamp for throat or nose work, the heating of a cautery, or even of lighting the area of an operation.

The knowledge acquired from the electrical devices of the motor-car can well be applied to the therapeutic instruments, and the object of this paper is to give a short account of the cases, which cropped up during one year in a general country practice, in which I thought electricity would be a useful therapeutic agent, and to consider where it was useful, and especially where it was not useful. I begin with cases treated with the constant current.

**Case 1.**—A male, aged about 30 years, who in his occupation as an insurance agent was exposed to all weathers. He came complaining of an acute neuritis of the right brachial plexus, which did not respond to anti-rheumatic remedies, so on June 21st he was treated with the constant current bath. The first bath relieved the pain, and after the twelfth sitting he was discharged cured.

**Case 2.**—Miss C., aged 44, a very stout lady, with a strong family history of rheumatic troubles, came complaining of pain and the formation of Heberden's nodes at the phalangeal joints of both hands. After two baths the pain was much relieved, and the nodules distinctly softened and lessened.

**Case 3.**—A lady sent me by the last patient because she had a similar condition of the hands. She had four baths and was improving nicely, when on the occasion of the fourth treatment she told me her feet were affected in the same way, and I suggested a combined foot and hand bath. This apparently did not please my patient, as the next day I received a note saying she did not desire further treatment.

The next two are cases of deafness with tinnitus. The treatment consisted of the application of the constant current by means of an electrode made from a binaural stethoscope, fitted with a binding screw, applied over the external meatus, as suggested by my friend, Dr. Lewis Jones, the indifferent electrode being put on the nape of the neck.

**Case 4.**—Miss C., aged 34, suffering for eighteen months from post-influenzal deafness. She can only hear when spoken to loudly, and complains very much of constant noises in the ears, as of escaping steam. She began with a dose of 3 *ma.* for twenty minutes, and the first dose convinced her that the treatment was useful. She did not attend very regularly, but came at intervals during nine months, having in all twenty-two treatments. The noises were kept to a bearable amount, and the hearing was much improved.

**Case 5.**—Mrs. H., complaining of deafness with tinnitus, which has existed for the last twenty years. No change visible in the drum membrane. The noises are very distressing. She was treated from March to July, altogether forty sittings. The dose was 3 *ma.* for twenty minutes. The noises were not benefited, and the deafness very little improved; a watch, which at first was only heard on contact with the ear, being at last heard at a distance of three inches. She grasped so at the hope of improvement that she constantly returned for treatment, although I tried to dissuade her. Eventually she concluded it was not worth while to continue. This case is interesting because there was a definite slight improvement after even twenty years of untreated disease, and because it shows how patient many deaf people are in carrying out any treatment which offers hope.

The next two are cases treated with the interrupted current.

**Case 6.**—A girl, aged 19 years, complaining of constant desire to micturate. She can control the bladder for a short time, but constantly has to empty it as the effort to control is so painful. She is a domestic servant, and has been obliged to leave her situation because of this trouble. After a course of iron and belladonna without any improvement, she was treated with the interrupted current. A conical electrode covered with wash leather was passed into the entrance of the vagina, and the other pad electrode was placed on the lumbar region. The strength of current used was such as, on trial, made the interosseous muscles of my own hand contract nicely. I may mention that these muscles make a very convenient test for the strength of either constant or interrupted current. After five sittings she was so much improved that she was able to attend divine service and sit it out without trouble, and she has continued to hold her water well ever since. The sittings were of fifteen to twenty minutes' duration.

**Case 7.**—A young man, aged 24 years, who complains of occasional nocturnal enuresis. This has existed since childhood, but has been specially troublesome lately. Drug treatment was tried without any effect, so treatment with the interrupted current was begun. One electrode was pressed on the perineum, and he lay with the other pressing on the lumbar region. After one or two sittings he improved, and treatment was not needed after the sixth application. He has had no bad attack since, though there is an occasional accident when out of sorts.

**Case 8** is again an interrupted current case, but of rather a different nature. She is a married lady, aged about 40 years. Has had a poor circulation all her life, and suffers badly from chilblains. Even in summer if she washes her hands in cold water the fingers go white, and remain dead for several hours. In October she was suffering from a chilblain. She was fitted out with an inexpensive coil driven by a dry cell, and shown how to connect this up to two small zinc electrodes. These were placed one in each of two basins of hot water, and she plunged a hand in either basin. As a result, the current ran nicely and smartly through each hand and arm. She was directed to withdraw the regulating tube until she had as strong a current as was pleasant, and to keep it running for twenty minutes at a time, and to use it every day. In a week the chilblain was well, and she kept her hands comfortable all through the winter.

This treatment of chilblains was suggested to me many years ago by my friend, Dr. Lewis Jones, who observed that the chilblains on the infantile paralytics who were treated at St. Bartholomew's with interrupted current baths quickly disappeared, and cases which I have treated myself have made me fully appreciate its value. It is necessary to begin before the chilblains break, as the application to a broken chilblain is apt to be painful. Still, even then I can strongly recommend it to anyone who suffers from this tiresome ailment ; if a very weak current be used the pain is quite bearable, and the ulcer soon heals, while the painful itching is quite allayed.

I now come to a series of cases which are to me the most interesting because they were treated with the static breeze generated by a Wimshurst machine.

I think the use of the static machine is unduly neglected in England. While I must confess to ignorance of the advantages of high-frequency current, as I have never worked with the apparatus, I feel sure that many of the cures claimed for high-frequency can be brought about by the static breeze.

**Cases 9 and 10** are so exactly alike that one account suffices. They were both elderly men suffering from a very chronic, but not very severe form of sciatica. They could both walk with the aid of a stick, and both needed help to step up on to the insulated stool. They were both treated in the same manner, viz. placed sitting on a stool on the insulated stool connected up to the negative pole of a four-plate Wimshurst machine ; then a metal pointer connected to the positive pole was passed up and down the limb at a distance just short of sparking, so that they got a brisk breeze. After ten minutes of this treatment the pointer was replaced by a ball of about two inches diameter, and a single spark, as long as the machine would give, was taken from any spot stated to be painful. Some four or six of such sparks were taken at each sitting.

It is necessary to be careful in this part of the treatment to make sure that only a single spark passes. One spark feels as if a sharp sudden tap with a hammer had been given to the selected spot. It startles, but does not really hurt. If, however, the operator is careless and allows a stream of sparks at this high tension to pass to or from his patient, it is extremely probable that he will not be called on for further treatment.

These two patients each stated that their pain was much relieved by the first treatment, and after the fourth sitting they had each discarded their walking-sticks, and were walking easily and comfortably.

**Case 11** was a man with sciatica, who had been helped but not quite cured by drug treatment. He received two doses of the static breeze, and that completed his cure.

**Case 12** was again a case of sciatica. A lady staying with friends in the district heard of the treatment of a friend suffering from sciatica by static breeze, and thought she would like to try the effect. There was only time for two sittings, but she was so satisfied that it did her good that on her return to London she obtained further treatment. Considering how unsatisfactory and tedious the treatment of a chronic sciatica often proves, there is here a useful field for static electricity.

**Case 13** was a woman, aged 45, who was broken down by overwork in nursing her sick mother. She had had a good deal of heavy lifting to do, and complained of an obstinate pain in the left sixth intercostal space. She was treated on two occasions with the breeze, but she was obviously afraid of the machine and averse to treatment, so it was stopped after the second sitting. It may safely be said that she received no benefit.

**Case 14** (also a visitor) was suffering from a neuritis of his brachial plexus. He too received only two treatments, and each treatment made him distinctly more comfortable. I have little doubt that had time allowed he would have been cured.

**Case 15,** an elderly gentleman suffering with a chronic arthritis of the right hip, and possibly also some trouble of the same sort in the spinal articulations, came to see if the breeze would relieve the constant pain in the back and leg. He was much pleased with the relief he got, and returned nine times for further treatment, coming twice or three times a week, and only left off attendance because he had to leave England. He certainly got some relief of pain, and walked better for a time after each sitting, but although he asserted that he got permanent good, I was never quite satisfied myself that he did. There was no doubt about the temporary good.

**Case 16** is, clinically, an interesting one. A young married woman, aged 30, married three years, came complaining of dysmenorrhœa and dyspareunia. At about twelve years she began to have pains in the back and stomach every month, but there was no sign of menstruation, until at twenty years of age she had such pain that some operation was done, presumably an operation for imperforate hymen. Since then the periods have been so painful that she always has to lie up one or two days. The pain caused by any attempt at coitus is so severe as to make her faint. Examination disclosed a large urethal caruncle, and an abnormally small vagina. Destruction of the caruncle with chromic acid rendered coitus practicable, but the dysmenorrhœa continued. Treatment with drugs and hot baths and douches did little good, so the static breeze was tried, and she was treated in all thirteen times. The pain was relieved so much as to allow her to get up and lie on a couch downstairs during the first two days of the period, instead of lying absolutely in bed, so that she received some benefit, but was not cured.

**Case 17.**—A young man, a visitor, was brought complaining of a constant pain in his back, the cause of which was not at all clear. Thinking it might be neuralgia, he was treated on three occasions with the static breeze. He received no benefit.

I now have to mention two cases in which treatment by ionisation was used.

**Case 18.**—The first, which I have already reported in the *Proceedings of the Electro-Therapeutic Society*, was a lady aged about 50, who has had a rodent ulcer since 1902. I cannot go very fully into her case, but may mention that she was treated with X-rays for some time without benefit, then under the influence of radium the ulcer was very nearly, but not quite, healed. Then, when it broke down again, it was treated by placing a pad of lint soaked in a 2 per cent. solution of zinc sulphate over the wound, and pressing a zinc electrode the size of the ulcer down on this. The zinc electrode was connected to the positive pole of the battery, and a current of 10 to 15 *ma.* was passed for ten minutes, or a few minutes longer if it could be borne. After many applications the ulcer was completely healed by July, 1906. In March, 1907, there was a slight breaking down, which healed again after one treatment with zinc, and we have gone on ever since with an occasional breaking down, which has healed up again after one or two treatments. She now has an ulcer about $\frac{1}{4}$-inch in diameter which shows healing edges.

This case gave me much encouragement to continue treatment of such cases by zinc ions, as when the treatment was begun the ulcer was quite 2 in. by $1\frac{1}{2}$ in. in size, and it was healed up for some months as the result of treatment.

**Case 19** was at first sight a similar case. A man, aged about 55, had an ulcer at the inner canthus of the right eye, which had existed for several months. Although rather doubtful of its nature, I tried to persuade myself that it was a rodent ulcer, and commenced zinc ionisation. He bore the pain of the treatment well, and had in all fourteen treatments. The ulcer never showed the white edge that a rodent does, and it showed no sign of healing under the treatment ; so it was excised, and was reported by the pathologist to be a squamous epithelioma. He reports : "In some respects it resembles rodent ulcer, in the rosetted appearance of the advancing edge and the small size of the epithelial cells at this part. It shows, however, cell nests and keratoid changes, so that it must be considered to be more malignant than rodent ulcer usually is."

This case is interesting because I was rendered suspicious of its nature, as it did not react to the zinc ions in the same way as the former case.

I may add that within the last fortnight he has consulted me for a recurrence of the growth. I am now treating it with X-rays.

**Case 20** was an old man with a large chronic ulcer of the leg. He had one or two sittings with zinc ionisation, in the hope that it might reduce the septicity of the wound. This did not, however, result. He was then X-rayed a few times, as just then that form of treatment had been recommended. He did not respond, and still remains with a large ulcer.

There remain three cases of malignant disease treated by X-rays. Of these two had their pain much relieved and the bulk of the growth reduced, but they eventually died of their cancer. The third, an old lady 82 years old, still goes on with a scirrhus mammæ. If she feels discomfort she comes and has a few treatments, which always result in a hardening and shrinking of the growth, and relief of any pain.

The last case was one in which malignant growth of the mamma was suspected. The lumps in the breast were treated by X-rays on several (seven) occasions without benefit. The growth was then excised, and found to be the result of chronic mastitis.

I have placed these cases on record because most of them were helped by very simple electrical treatment. When the diversity of the symptoms relieved is considered, I hope I shall have established my contention that electricity is a very useful therapeutic agent to the general practitioner.

